# Outcome of bleomycin electrosclerotherapy of slow-flow malformations in adults and children

**DOI:** 10.1007/s00330-024-10723-6

**Published:** 2024-04-16

**Authors:** Vanessa F. Schmidt, Özlem Cangir, Lutz Meyer, Constantin Goldann, Susanne Hengst, Richard Brill, Susanne von der Heydt, Milton Waner, Daniel Puhr-Westerheide, Osman Öcal, Muzaffer Reha Ümütlü, Nabeel Mansour, Jan Rudolph, Alena Sint, Florian Obereisenbuchner, Beate Häberle, Jens Ricke, Max Seidensticker, Walter A. Wohlgemuth, Moritz Wildgruber

**Affiliations:** 1 Department of Radiology, LMU University Hospital, LMU Munich, München, Germany; 2 Interdisziplinäres Zentrum für Gefäßanomalien (IZGA), LMU University Hospital, LMU Munich, München, Germany; 3 Department of Pediatric Surgery, Center for Vascular Malformations, Klinikum Barnim GmbH, Werner Forssmann Hospital, Eberswalde, Germany; 4 Clinic and Policlinic of Radiology, Martin-Luther University Halle-Wittenberg, Halle (Saale), Germany; 5 Department of Radiology, Center for Vascular Malformations, Klinikum Barnim GmbH, Werner Forssmann Hospital, Eberswalde, Germany; 6 Vascular Birthmark Institute of New York, New York, NY USA; 7 Department for Pediatric Surgery, LMU University Hospital, LMU Munich, München, Germany

**Keywords:** BEST, Electro-chemotherapy, Sclerotherapy, Bleomycin, Slow-flow vascular malformations

## Abstract

**Objectives:**

To evaluate the safety and clinical outcome of bleomycin electrosclerotherapy (BEST) for treating extracranial slow-flow malformations.

**Methods:**

In this retrospective investigation of a multicenter cohort presenting symptomatic slow-flow malformations, patient records were analyzed with respect to procedural details and complications. A treatment-specific, patient-reported questionnaire was additionally evaluated, obtained 3–12 months after the last treatment, to assess the subjective outcomes, including mobility, aesthetic aspects, and pain, as well as the occurrence of postprocedural skin hyperpigmentation. All outcome parameters were compared according to patients’ age.

**Results:**

Overall, 325 BEST treatments were performed in 233 patients after intralesional and/or intravenous bleomycin injection. The total complication rate was 10.2% (33/325), including 29/352 (8.9%) major complications. Patient-reported mobility decreased in 10/133 (8.8%), was stable in 30/113 (26.5%), improved in 48/113 (42.5%), and was rated symptom-free in 25/113 (22.1%) patients. Aesthetic aspects were rated impaired compared to baseline in 19/113 (16.8%), stable in 21/133 (18.6%), improved in 62/113 (54.9%), and perfect in 11/133 (9.7%) patients. Postprocedural skin hyperpigmentation occurred in 78/113 (69%) patients, remaining unchanged in 24/78 (30.8%), reduced in 51/78 (65.5%), and completely resolved in 3/78 (3.8%) patients. The median VAS pain scale was 4.0 (0–10) preprocedural and 2.0 (0–9) postprocedural. Children/adolescents performed significantly better in all parameters compared to adults (≥ 16 years) (mobility, *p* = 0.011; aesthetic aspects, *p* < 0.001; pain, *p* < 0.001).

**Conclusions:**

BEST is effective for treating slow-flow vascular malformations, with few but potentially significant major complications. Regarding patient-reported outcomes, children seem to benefit better compared to older patients, suggesting that BEST should not be restricted to adults.

**Clinical relevance statement:**

Bleomycin electrosclerotherapy is a safe and effective approach and therapy should not be restricted to adults due to good clinical outcomes in children.

## Key Points

- *In this multicenter study investigating 325 bleomycin electrosclerotherapies, high subjective response in mobility, aesthetic aspects, and pain was reported, accompanied by few but potentially severe complications.*
- *Skin hyperpigmentation after treatment is the most common side effect, which frequently fades in the postprocedural course.*
- *Children/adolescents (0–15 years) performed significantly better in all outcome parameters regarding mobility, aesthetic aspects, and pain, when compared to adults.*

## Introduction

According to the classification of the International Society for the Study of Vascular Anomalies (ISSVA) [1], vascular malformations are divided into slow-flow and fast-flow lesions, related to the underlying flow pattern, which influences both treatment decisions and prognosis [2–5]. Among slow-flow malformations, venous and lymphatic malformations (VMs and LMs) represent disease patterns that frequently become apparent in childhood [6] and may severely limit the patient’s quality of life with symptoms such as pain, swelling, and aesthetic and functional impairments [7]. Surgery in slow-flow malformations is frequently limited to small, circumscribed lesions or to debulking in case of large volumes being affected [8]. Sclerotherapy with different sclerosing agents is the most commonly used minimally invasive treatment to treat dysplastic vasculature via an inflammatory reaction and to significantly improve patients’ symptoms [8]. Bleomycin, which initially became known due to its cytotoxic and antibiotic effects, is one of the most frequently used off-label sclerosing agents for slow-flow malformations worldwide [9]. However, due to the charged and large-molecule structure, its effectiveness is limited, and some of the patients with slow-flow malformations present no or diminished clinical response after repeated sclerotherapy sessions [10]. Recently, a therapeutic method has been developed that promises to increase the efficacy of bleomycin. Using reversible electroporation, the application of short high-voltage electrical impulses leads to a reversible local increase in cellular membrane permeability, whereby the intracellular concentration of bleomycin and its local effectivity rise by a factor of up to several thousand-fold [11]. A selective effect on vascular endothelial cells may augment the effectiveness of reversible electroporation, thus making it a promising treatment method, particularly in therapy-resistant vascular malformations as well as lesions where, due to the large extent, conventional sclerotherapy is unlikely to achieve a clinical success within a reasonable number of treatment cycles [12, 13]. Besides a few recently published small case series, no larger data exist regarding the safety and efficiency of the combination of reversible electroporation and bleomycin in treatment of slow-flow malformations [14–18]. Therefore, we analyzed this treatment modality in a larger multicenter cohort of patients with slow-flow malformations including reported patient outcomes, in both adults and children.

## Methods

### Study design

In this retrospective, multicenter study of three vascular anomalies, consecutive patients with symptomatic slow-flow vascular malformations (simple and combined), according to ISSVA [1], treated with bleomycin electrosclerotherapy (BEST) between October 2020 and July 2023 were included. The study was approved by the local ethics committee (Protocol No. 23-0035, 01/16/2021) and was performed following the 1964 Helsinki declaration and its later amendments. Data collection was performed using electronic patient records as well as the Picture Archiving and Communication System. Diagnosis, based on patient history, ultrasound and magnetic resonance (MR) imaging, and clinical examination including indication for electrosclerotherapy, was established on the basis of interdisciplinary consensus at three interdisciplinary vascular anomaly centers in Germany. Interdisciplinary vascular board decisions for BEST were made following discussions among at least one interventional radiologist, a pediatrician/pediatric surgeon in case of the patient being < 18 years old, as well as at least one physician specialized in surgery of vascular anomalies. Additional specialties such as hemato-oncology, maxillofacial surgery, head and neck surgery, orthopaedic surgery, plastic surgery, and hemostaseology were included in the discussion as needed. The indications for BEST were pain, swelling, bleeding, recurring infections, repetitive thrombosis, consumptive coagulopathy, aesthetic disfigurement, and functional impairment. Patients were not excluded due to previous minimally invasive or surgical treatments if there was a therapy-free interval of six months before the first BEST procedure. In general, BEST was not performed in breastfeeding or pregnant women, patients with childbearing potential not using contraception, patients with intolerance to bleomycin or previous bleomycin-related toxicity, patients who already received a cumulative dose of bleomycin of 100 mg or more, patients with chronic pulmonary dysfunction, patients with previous chest radiation therapy, and patients with a history of epilepsy/seizures [19, 20].

### Bleomycin electrosclerotherapy

Interventional treatment was performed under general anesthesia. In case of complex lesions as well as unclear venous drainage, direct percutaneous injection of the contrast agent into the malformation under fluoroscopy guidance was performed. Intravenous or intralesional injection of bleomycin was followed by electrode positioning and subsequent application of reversible electroporation pulses. Specific electrode design (hexagonal electrodes, finger electrodes, and freely positionable needle electrodes) was chosen in relation to lesion size and localization as well as tissue composition. Needles were placed within the margins of the malformations at distances ranging from 0.5 to 3 cm. Thus, if technically possible, the target volume was completely covered with repetitive punctures, with relevant gaps or overlaps being avoided. Large lesions in deep locations were treated with different numbers of needle electrodes, allowing for free positioning in variable geometries. The maximum overall bleomycin dose (intralesional and/or intravenous) allowed was 0.2 mg per kg bodyweight per treatment session and less than 1 mg/kg bodyweight cumulative according to the standard bleomycin sclerotherapy, which is less than that in previous reports [19]. To apply the reversible electric pulses, the electroporation system (Cliniporator™ VITAE, IGEA S.p.A., Carpi, Italy) was used, which provides several independently controlled and isolated outputs, each reaching up to 3000 V (maximum current: 50 A). Consequently, electrical impulses with a duration of 100 μs between each pair of electrodes were generated. The electroporation was performed immediately after intralesional bleomycin application or 8 min after starting intravenous bleomycin application.

### Follow-up

In the three centers involved, patients were scheduled for a standardized follow-up regime. The first clinical follow-up was performed 1–3 months after each BEST session. In case of insufficient improvement of symptoms and residually perfused lesion(s) being present, an additional BEST was performed.

### Treatment-specific, patient-reported questionnaire

For follow-up evaluation of subjective symptom assessment, a treatment-specific, patient-reported questionnaire (see Supplementary Appendix 1) was analyzed, which was sent out to patients between three and 12 months after (the last) treatment, to subjectively assess the patients’ outcomes in mobility, aesthetic aspects, and pain as well as postprocedural skin hyperpigmentation/discoloration. The patient-reported outcomes in mobility were measured using the following grading scale: decreased compared to baseline, stable, improved, and symptom-free. The patients’ outcomes in aesthetic aspects were classified into the following four categories: impaired compared to baseline, stable, improved, and perfect. The occurrence of hyperpigmentation was recorded dichotomous by the categories *yes/no*. In case postprocedural skin hyperpigmentation was present, the further course of this alteration was recorded by the patients via the following classification: unchanged, reduced, and completely resolved. A 10-point visual analogue scale (VAS) was used to assess pain symptoms at two time points, preprocedural and postprocedural. In a second step, all outcome parameters were compared related to the median age of the patients. Therefore, two groups based on the median age of the cohort (15 years), children/adolescents (0–15 years) and adults (≥ 16 years), were determined, and two similarly sized groups could be formed. In addition, all outcome parameters were compared between pretreated and treatment-naive patients, as well as according to lesion extent (one involved anatomical area vs. extensive lesions with ≥ 2 anatomical areas involved).

### Peri- and postprocedural complications

All documented peri- and postprocedural complications were classified into minor and major adverse events (AE) [21]. Special attention was paid to document skin alterations (e.g., skin hyperpigmentation/discoloration, necrosis, blebs) and peripheral nerve injuries (e.g., paresis, impairment of sensibility).

### Statistical analysis

Descriptive statistics were used to analyze the distribution of patients among the different categories. Kolmogorov–Smirnov (K–S) test was used for the assessment of normality. Data are presented as mean (±standard deviation) or as median (range, minimum–maximum). Subanalyses were performed using the Pearson’s chi-squared test for categorical data and the Mann–Whitney *U* test for metric data. Statistical testing was conducted using SPSS (version 26.0, IBM Corp., USA); *p* < 0.05 was considered significant.

## Results

### Patient characteristics

A total of 233 consecutive patients, 118 males and 115 females, with symptomatic, extracranial, slow-flow vascular malformations underwent a total of 325 BEST treatments (Table 1). The median age was 13.0 years (range, 0–71 years) at the time of the first treatment. A total of 163/233 (70.0%) patients received one procedure, 52/233 (22.3%) two procedures, and 18/233 (7.7%) three or more procedures; see Table 1. In general, the 233 patients with slow-flow lesions presented in 161/233 cases (69.1%) with simple VMs, in 54/233 (23.2%) with simple LMs, in 7/233 (3.0%) with combined veno-lymphatic malformations (VLMs), in 7/233 (3.0%) with combined capillary-venous malformations, in 3/233 (1.3%) with combined capillary-veno-lymphatic malformations, and in 1/233 (0.4%) with combined capillary-lymphatic malformation. The involvement of anatomical areas was present as follows: the head/neck areas in 101/233 (43.3%) cases, the lower extremities in 96/233 (41.2%) cases, the upper extremities in 29/233 (12.4%) cases, and the trunk/buttock area in 46/233 (19.7%) cases. Thereby, 33/233 (14.2%) patients presented with extensive lesions expanding into more than one of these anatomical areas. For further details, see Table 1. Both therapy-naive patients (187/233, 80.3%) and patients having undergone previous invasive treatments (46/233, 19.7%) by debulking surgery (6/233, 2.6%) or sclerotherapy (42/233, 18.0%) without sufficient symptom improvement were included.

**Table 1:** Patient characteristics of the study cohort

Characteristic	Cohort (total, *n* = 233)
Age at treatment, median (range)	13.0 (0–71)
Men	118 (50.6%)
Slow-flow vascular malformations	233/233 (100%)
Simple malformations	216/233 (92.7%)
VMs	161/233 (69.1%)
LMs	54/233 (23.2%)
Combined malformations	18/233 (7.7%)
VLMs	7/233 (3.0%)
CVMs	7/233 (3.0%)
CLMs	1/233 (0.4%)
CVLMs	3/233 (1.3%)
Involved anatomical areas	
Head/neck	101/233 (43.3%)
Lower extremity	96/233 (41.2%)
Upper extremity	29/233 (12.4%)
Trunk/buttocks	46/233 (19.67%)
Extensive lesions^a^	33/233 (14.2%)
2 areas	27/233 (11.6%)
3 areas	5/233 (2.1%)
4 areas	1/233 (0.4%)
Previous invasive treatments	46/233 (19.7%)
Debulking surgery only	4/233 (1.7%)
Sclerotherapy only	40/233 (17.2%)
Both	2/233 (0.9%)

*CLM* capillary-lymphatic malformation, *CM* capillary malformation, *CVLM* capillary-veno-lymphatic malformation, *LM* lymphatic malformation, *VLM*veno-lymphatic malformation, *VM* venous malformation

^a^ Vascular malformations extending to more than one anatomical area

### Procedural characteristics

**Fig. 1:**
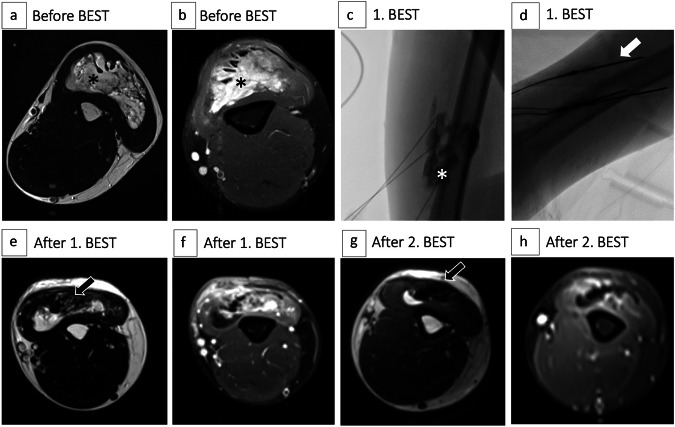
27-year-old male patient with intramuscular venous malformation (VM) of the upper extremity undergoing two subsequent bleomycin electrosclerotherapies (BESTs). **a**, **b** T2-weighted (T2w) and fat-suppressed (fs) T1-weighted (T1w) axial magnetic resonance (MR) images prior to treatment with BEST showing the extent of a symptomatic VM (asterisks) predominantly located in the triceps brachii muscle. **c**, **d** Digital subtraction angiography (DSA) images during first BEST session. The asterisk shows the filling of the dysplastic venous structures of the lesion after KM insertion. The arrow mark shows the parallelly inserted needle electrodes during therapy. **e**, **f** T2w and fs T1w axial MR images three months after the first BEST session revealing significant regredience of the vascularized parts of the VM (arrow) with accompanying improvement in the patient’s pain symptoms. **g**, **h** T2w and fs T1w axial MR images three months after the second BEST revealing near-complete resolution of the malformation. The arrow mark shows the volume-reduced lesion

The mean number of BESTs per patient was 1.4 (± 0.7); for further details, see Table 2. Thereby, 235/325 (72.3%) procedures were performed in children (< 18 years). Finger electrodes were primarily used in 223/325 (68.6%) procedures, while in 78/325 (24.0%) and 24/325 (7.4%) treatments hexagonal electrodes and freely positionable needle electrodes were predominantly applied. In 38/325 (11.7%) cases a switch to another electrode geometry was performed. The mean number of electroporation cycles per treatment was 44.0 (± 42.3). Among the 325 BESTs, in 308/325 (94.8%) cases bleomycin was applied intralesionally (see Fig. 1), with a mean dose of 4.0 (± 3.2 mg) per session; in 32/325 (9.8%) cases bleomycin was applied locally into the veins, with a mean dose of 6.6 mg (± 4.7 mg) per session. Thus, in 10/325 (3.1%) treatments, patients received both intravenous and intralesional bleomycin application, with an overall dose of bleomycin of 7.1 mg (± 4.9 mg) per session (Table 2).

**Table 2:** Procedural data of the study

Characteristic	Cohort (total, *n* = 233)	BESTs (total, *n* = 325)
BESTs per patient, mean (±SD)	1.4 (± 0.7)	
Total BESTs per patient		
1	163/233 (70.0%)	
2	52/233 (22.3%)	
3	15/233 (6.4%)	
4	2/233 (0.9%)	
5	1/233 (0.4%)	
Primarily used electrode		
Finger		223/325 (68.6%)
Hexagonal		78/325 (24.0%)
Needle		24/325 (7.4%)
Dose (mg) of bleomycin		
Intralesional (*n* = 308), median (range)		3.0 (0.5–25)
Intravenous (*n* = 32), median (range)		5.0 (1–15)
Both (*n* = 10), median (range)		6.0 (4–21)
Cycles of electroporation, median (range)		30 (1–245)

*BEST* bleomycin electrosclerotherapy, *SD* standard deviation

### Safety and complications

Postprocedural skin hyperpigmentation/discoloration was evaluated on the basis of the treatment-specific patient-reported questionnaires, see the section “Patient-reported outcome”.

Besides, minor and major complications were reported after 33/325 (10.2%) BESTs (grade 1–4) consisting of 2/325 (0.6%) periprocedural and 31/325 (9.5%) postprocedural events, the latter including 8/325 (0.9%) late complications.

In detail, the two periprocedural complications included 1/325 (0.3%) mucosal hemorrhage during treatment of an oral lesion, which had to be sutured with Vicryl 3-0 (grade 1). Another periprocedural event (1/325, 0.3%) occurred after BEST of a lower limb VM, in proximity to the popliteal artery. Right after applying the BEST cycles sudden paleness of the lower extremity occurred, which, following subsequent intraarterial angiography, turned out to be caused by massive spasm of the artery. Extensive spasmolysis using nitroglycerin, calcium blockers, and prostaglandin E1 intraarterially resolved the massive spasm, which persisted over several hours (grade 3).

**Fig. 2:**
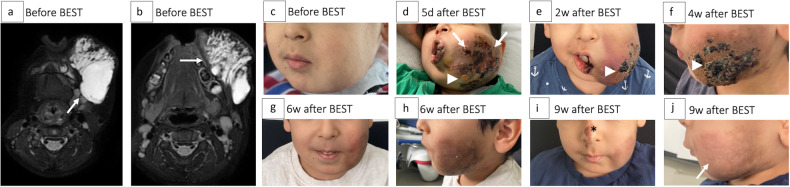
Four-year-old male patient with subcutaneous lymphatic malformation (LM) of the left cheek undergoing one bleomycin electrosclerotherapy (BEST). **a**, **b** T2-weighted, axial magnetic resonance images prior to treatment showing a partially micro- and partially macrocystic LM (arrows). **c** Clinical presentation of the patient prior to treatment including noticeable swelling of the affected region with consecutive mouth asymmetry. **d** Clinical presentation of the patient five days after BEST with extended postinterventional swelling (arrows) with consecutive dyspnea, which was strictly monitored, as well as skin alterations such as hyperpigmentation, necroses, and blebs (arrowhead). As oral food intake was not possible, the patient was fed intravenously, and prednisolone was administered. **e**–**h** The decreasing swelling and good healing of necrosis and blebs over time (arrowheads) while the distinct hyperpigmentation on the treated skin was pronounced on the maximum. **i**, **j** Significant reduction of skin hyperpigmentation six weeks after BEST (arrow) compared to (**h**)

The 31 postprocedural complications included local skin necroses/blebs after 15/325 treatments (4.6%, all grade 3) at the treated area, see Fig. 2. Of these, 7/325 (2.2%) became superinfected, including 3/325 (0.9%) abscess formations; all 15 AEs were conservatively resolved by intensified wound management during prolonged hospitalization, whereby the abscess formations were additionally treated with antibiotics. Further postprocedural AEs involved prolonged or excessive swelling at the injection site (6/325, 1.8%, grade 2–3); partially entailing elongated postprocedural observation (3/325, 0.9%, grade 2); partially additional measures that were necessary, such as prednisolone administration, intravenous feeding, or intensive monitoring due to dyspnea (3/325, 0.9%). In 1/325 case (0.3%), a persistent leg paresis occurred immediately after the procedure due to needle puncture injury of the sciatic nerve during the treatment of a deep lesion of the gluteal area. Furthermore, after 1/325 (0.3%) treatment of an extensive LM, the patient developed significant symptoms (worsening of the general condition, emesis, pronounced hematoma, and swelling in treated areas) due to disseminated intravascular coagulation, which presented with anemia and thrombocytopenia in the laboratory. Several transfusions (electrocytes, platelets, fibrinogen, antithrombin-3, fresh frozen plasma, prothrombin complex concentrate), concomitant administration of tranexamic acid, heparin, and prednisolone, antiemetic (ondansetron, dimenhydrinate) and pain medication (piritramide, metamizole), and compression bandages of the treated areas did not lead to an immediate improvement. As the patient progressed to abdominal compartment syndrome (up to 29 mmHg) and acute renal failure with anuria and without any response to furosemide, the patient was transferred to an intensive care unit. Here, complete control and resolution of all symptoms could be achieved without permanent sequelae (grade 3). After 8/325 (0.9%) BESTs, significant scar formation occurred that did not resolve > 30 days postoperatively (late complication, grade 4).

Overall, the major complication rate (grade > 2) was 29/325 (8.9%). In our patient cohort, there were no allergic reactions due to administered drugs, postinterventional syncope, or nausea/vomiting. Additionally, there were no pulmonary complications immediately or during follow-up.

### Patient-reported outcome

The treatment-specific, patient-reported questionnaire was completed by 113/233 (48.5%) patients. Both in mobility and aesthetic aspects, the outcome was mostly rated as improved (48/113, 42.5%; 62/113, 54.9%); further details are presented in Table 3. Postprocedural skin hyperpigmentation/discoloration occurred in 78/113 (69.0%) patients. In these 78 cases, the further course of the skin alterations was reported as unchanged by 24/78 (30.8%, 21.2% of total) patients, as reduced by 51/78 (65.5%, 45.1% of total), and as completely resolved by 3/78 (3.8%, 2.6% of total). The median preprocedural VAS pain scale was rated as 4.0 (0–10) and that at follow-up as 2.0 (0–9). Thus, when comparing the pre- and postprocedural VAS pain scales, the scale was reduced in 47/113 (41.6%) patients and even regressed completely in 20/113 (17.7%) patients (to VAS 0); for more details, see Table 3.

**Table 3:** Comparison of outcomes according to patients’ age

Characteristic	Total cohort (*n* = 113)	Ped./adol. group^a^ (*n* = 62)	Adult group^b^ (*n* = 51)	*p*-Value
Outcome in mobility				0.011^c^
Decreased	10/113 (8.8%)	4/62 (6.5%)	6/51 (11.8%)	
Stable	30/113 (26.5%)	14/62 (22.6%)	16/51 (31.4%)	
Improved	48/113 (42.5%)	23/62 (37.1%)	25/51 (49.0%)	
Symptom-free	25/113 (22.1%)	21/62 (33.9%)	4/51 (7.8%)	
Outcome in aesthetic aspects				<0.001^c^
Impaired	19/113 (16.8%)	4/62 (6.5%)	15/51 (29.4%)	
Stable	21/113 (18.6%)	9/62 (14.5%)	12/51 (23.5%)	
Improved	62/113 (54.9%)	38/62 (61.3%)	24/51 (47.1%)	
Perfect	11/113 (9.7%)	11/62 (17.7%)	0/51 (0.0%)	
Pain VAS score preprocedural, median (range)	4.0 (0–10)	3 (0–10)	5 (0–9)	0.012^d^
Pain VAS score postprocedural, median (range)	2.0 (0–9)	0 (0–8)	3 (0–10)	<0.001^d^
Pain VAS score pre- to postprocedural				<0.001^c^
Increased	13/113 (11.5%)	2/62 (3.2%)	11/52 (21.2%)	
Stable	12/113 (10.6%)	6/62 (9.7%)	6/52 (11.5%)	
Reduced	47/113 (41.6%)	18/62 (29.0%)	29/52 (55.8%)	
Completely regressive	20/113 (17.7%)	17/62 (27.4%)	3/52 (5.8%)	
Pain-free before and after BEST	21/113 (18.6%)	19/62 (30.6%)	2/52 (3.8%)	

*Adol*. adolescent, *Ped.* pediatric, *VAS* visual analogue scale

^a^ Pediatric/adolescent group, 0–15 years

^b^ Adult group, ≥ 16 years

^c^ Pearson’s chi-squared

^d^ Mann–Whitney *U* test

Interestingly, the comparison of the patient-reported outcome according to the patient’s age at treatment revealed significant differences. The outcome in mobility was significantly different between the pediatric/adolescent and adult group (Pearson’s chi-squared test, *p* = 0.011); exemplarily, 21/62 (33.9%) children/adolescents rated symptom-free, while 4/51 (7.8%) adults did so; for further details, see Table 3. The outcome related to aesthetic aspects was significantly different between both groups (Pearson’s chi-squared test, *p* < 0.001); exemplarily, only 4/62 (6.5%) children/adolescents reported an aesthetical decrease, while 15/51 (29.4%) adults did so; for further details, see Table 3. When comparing the pre- to postprocedural VAS pain scale between the pediatric/adolescent and adult groups, there were significant differences as well (Pearson’s chi-squared test, *p* < 0.001). Thus, 17/62 (27.4%) children/adolescents presented complete regression, while only 4/52 (5.8%) adults did so. Only 2/62 (3.2%) children/adolescents reported increased pain, while 11/52 (21.2%) adults did so (see Table 3).

The comparison of pretreated and therapy-naive patients revealed significant differences regarding the outcome in mobility as well (Pearson’s chi-squared test, *p* = 0.011); see Supplemental Table 1. When comparing the pre- to postprocedural VAS pain scale, there were no significant differences (Pearson’s chi-squared test, *p* = 0.118), though the postprocedural VAS score differed between both groups (pretreated: median 3, 0–9 vs. therapy-naive: median 1, 0–10; *p* = 0.005), see Supplemental Table 1. The comparison according to the lesion extent revealed no significant differences when one area only was affected compared to two or more areas affected; the detailed results are presented in Supplemental Table 2.

## Discussion

In this study of BEST in slow-flow vascular malformations, a positive subjective response in mobility, aesthetic aspects, and pain in the majority of patients treated is being reported, accompanied by few but potentially severe complications. Skin hyperpigmentation after treatment is the most common side effect but frequently fades in the postprocedural course. With respect to patient-reported outcomes, children seem to benefit more than older patients. As BEST is a novel therapeutic approach for vascular malformations, its effectiveness and safety have only been reported in few single-center cohorts so far [14–18]. Thus, this study provides information on safety and patient-reported outcomes, as well as initial data for patient selection, from a larger cohort for the first time.

Initially, reversible electroporation in combination with intralesional or intravenous application of bleomycin was established in the invasive treatment of skin tumors and bleeding of cutaneous metastases, such as malignant melanoma or otolaryngological tumors [22–24]. Here it was observed that BEST exerts dedicated vascular effects, such as a vascular lock and vascular disrupting effects [25]. Regarding vascular malformations, intralesional injection of different types of sclerosing agents was established as standard treatment for vascular malformations [9]. Bleomycin is one of the most frequently used sclerotherapy agents treating these lesions, but repeated procedures are routinely required to achieve sufficient response [10]. Therefore, the combination with short electric pulses may improve the effectiveness of bleomycin as it increases the intracellular drug uptake into the endothelial lining of the affected vasculature [20]. In vascular malformations, blood vessels present atypically, and endothelial cell proliferation is higher compared to normal blood vessels [26], similar to the changes seen in tumors. This may support that BEST can be effective in both. In addition to reduced number of treatment sessions, particularly in extensive lesions, the mechanism of BEST also offers novel possibilities for microcystic LM that at present are difficult to treat with conventional sclerotherapy [27]. It may be further developed as a potential tool for facial vascular malformations by extending the treatment indications for lesions with a high risk of necrosis or peripheral nerve injury using alcohol-based sclerosing agents [28].

Reports on BEST are still limited, consisting of few case series published [14–18]. In summary, different types of vascular malformations, including slow- and fast-flow lesions, have been treated, with promising clinical results in these small cohorts. While lesion size was significantly reduced, the number of treatments required was much lower when performing BEST compared to when conventional bleomycin sclerotherapy was used alone. Wohlgemuth et al. [14] reported a median therapy-induced volume reduction of 86%, and most patients required only one treatment session. Intralesional bleomycin was applied in most cases, either mixed with lidocaine or diluted. Bleomycin dosage varied between reports, but it was generally lower compared to traditional sclerotherapy [14, 18, 20]. In the cases reported to date, the most common AEs have been skin hyperpigmentation, necroses, blebs, and local superinfection [14, 18].

Due to the presence of mixed entities (combined slow-flow malformations in different locations) in our cohort, this study is difficult to compare with cohorts using other treatment approaches. This study provides treatment-related findings after BEST and focused on safety and patient-reported outcomes and is based on a large cohort that includes both adults and pediatric patients. The overall major complication rate in this large cohort was low, confirming the initial results of case series that it is a safe treatment approach in vascular malformations when applied correctly [20]. Similar to traditional sclerotherapy, the most common complications remain localized at the treated area and include necrosis, blebs, and, rarely, superinfection. However, the reported ratio of major complications to the overall complication rate demonstrates that potentially severe complications can occur and possibly lead to permanent impairment. Based on the patient-reported outcomes from our three centers, initial skin hyperpigmentation occurs in about half of the patients. Even if these regularly fade in the postprocedural course, the patient should be informed and consent to potentially permanent local hyperpigmentation. The effectiveness of BEST was demonstrated by the patient-reported outcome regarding mobility and aesthetic aspects, as in two-thirds of the questionnaires improvement or a symptom-free/perfect result after therapy was reported. However, it should be considered that a high proportion of head and neck localizations were present in this cohort, which predominantly do not/slightly cause mobility impairment. The reported patient satisfaction after treatment supports the value of this novel approach, particularly as it may result in increasing patients’ participation in everyday life. The ‘per-procedure’ costs of BEST clearly exceed the costs of a conventional sclerotherapy session, but we assume that in the end this may result in similar total costs per patient due to the higher effectiveness and fewer treatment cycles required when BEST is being used; however, further studies on cost-effectiveness are needed in the future.

Prospective studies investigating the patients’ health-related quality of life following BEST are still needed [29]. The reported outcomes of pediatric and adolescent patients (0–15 years) are particularly interesting. Even if children may initially present with less severe symptoms before BEST, they may perform significantly better with respect to subjective outcome. This offers the potential to control the vascular lesions and their progression already at a young age, with the patients remaining either with improved symptoms or symptom-free/-poor during adolescence. This may be further substantiated due to the finding of this study that in the group of adults responding well overall, there are a few patients who reported an even worse outcome after treatment compared to before BEST. Patients who had previously undergone invasive treatment also performed significantly worse compared to treatment-naive patients, although this may also be biased due to the patients’ age, which was higher in the pretreated group. Thus, proper patient selection is of major importance for electrosclerotherapy and needs to be addressed in subsequent studies.

### Limitations

The limitations of the study include the retrospective character of the study including a moderate response rate to the patient questionnaire, which may include a certain bias. The reasons for moderate patient feedback include the fact that a substantial amount of non-native speaking patients from non-foreign countries were treated in the dedicated specialized centers within the cohort, who did not reply during follow-up. Moreover, the patients’ questionnaire was not properly validated psychometrically, as all existing validated health-related quality-of-life assessments are not designed to specifically evaluate BEST. A certain bias induced by differences in reply between children and adults has to be assumed. Children in general suffer from malformation-associated symptoms for a shorter time interval, while adults frequently present with an already chronic pain syndrome. The different subjective response may thus be a summary effect containing a potential bias, as described above. In addition, no systematic MR imaging was performed in this cohort at follow-up, so an imaging-based objective therapy response was not evaluated. Despite these limitations, the large cohort size being investigated in a real-world scenario provides valid clinical results for BEST as a new treatment option for vascular malformations, especially for those ranging from difficult to impossible-to-treat until now.

### Conclusions

As a new technology for treatment of slow-flow vascular malformations, BEST is effective in a variety of lesion locations. Patients should be aware of common skin hyperpigmentation after treatment, in most cases fading at least partially in the postprocedural course and having few but potentially severe complications. Young patients in particular improve substantially, suggesting that the treatment should not be restricted to adults.

## Supplementary information

Supplementary Information

## Abbreviations

AE: Adverse event
BEST: Bleomycin electrosclerotherapy
ISSVA: International Society for the Study of Vascular Anomalies
LMs: Lymphatic malformations
MR: Magnetic resonance
VAS: Visual analogue scale
VMs: Venous malformations
