# Supplementary material for: Outcome of bleomycin electrosclerotherapy of slow-flow malformations in adults and children
Source: Eur Radiol. 2024 Apr 16;34(10):6425–34. doi: 10.1007/s00330-024-10723-6 (PMC11399160; doi:10.1007/s00330-024-10723-6)
Supplement: Supplementary file 1 — Supplementary Information [file 330_2024_10723_MOESM1_ESM.pdf]

Outcome of Bleomycin Electrosclerotherapy of Slow-Flow Malformations in Adults and Children

ELECTRONIC SUPPLEMENTARY MATERIAL

Eur Radiol (2024) Schmidt VF, Cangir Ö, Goldann C, et al.

Questionnaire after Bleomycin-Electro-Sclerotherapy

Name:

Date of birth:

Today's date:

1. Treatment-specific outcome

Outcome in mobility (compared to baseline)

- ☐ symptom-free
- ☐ improved
- ☐ stable
- ☐ decreased

Outcome in aesthetic aspects (compared to baseline)

- ☐ perfect
- ☐ improved
- ☐ stable
- ☐ impaired

Hyperpigmentation/Discoloration after treatment

- ☐ yes
- ☐ no

If yes: Further course

- ☐ unchanged
- ☐ reduced
- ☐ completely resolved

2. **Pain -** Visual analogue scale (VAS) **before treatment***:

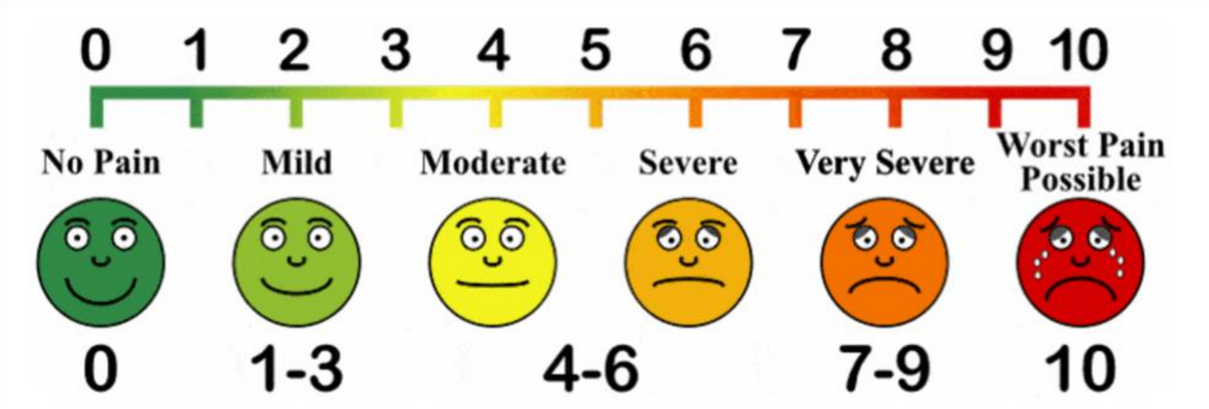

3. **Pain -** Visual analogue scale (VAS) **now (3-12 months after treatment)***:

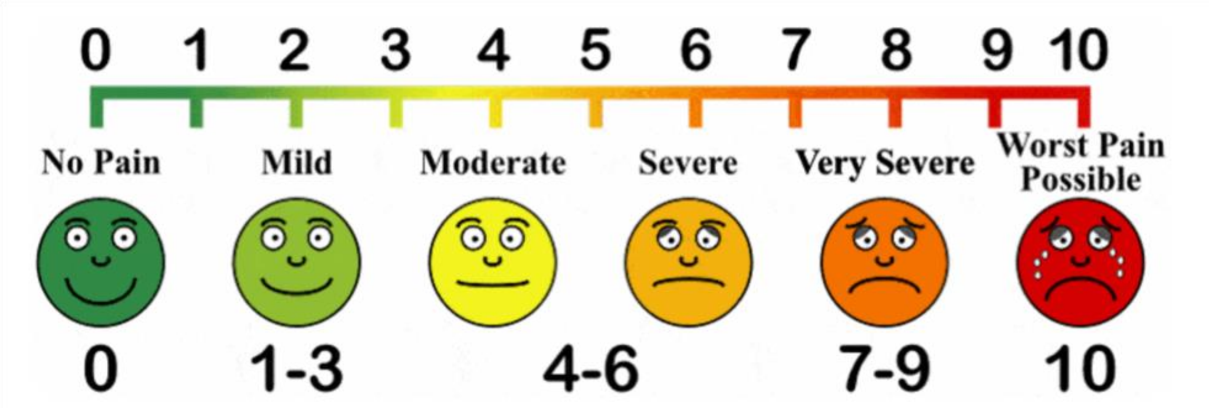

* Please mark/check the pain intensity. **0**= no pain at all to **10**= strongest pain.

Supplemental Table 1. Comparison of Outcome according to Pre-treated and Therapy-naive Patients.

Characteristic	*Total cohort (n=113)*	*Previous invasive treatment(s) (n=38)*	*Therapy-naiv patients (n=75)*	*p-value*
Outcome in mobility				**p=0.011**
Decreased	10/113 (8.8%)	7/38 (18.4%)	3/75 (4.0%)	
Stable	30/113 (26.5%)	13/38 (34.2%	17/75 (22.7%	
Improved	48/113 (42.5%)	14/38 (36.8%	34/75 (45.3%	
Symptom-free	25/113 (22.1%)	4/38 (10.5%)	21/75 (28.0%	
Outcome in aesthetic aspects				p=0.059‡
Impaired	19/113 (16.8%)	11/38 (28.9%	8/75 (10.7%)	
Stable	21/113 (18.6%)	8/38 (21.1%)	13/75 (17.3%	
Improved	62/113 (54.9%)	17/38 (44.7%	45/75 (60.0%	
Perfect	11/113 (9.7%)	2/38 (5.3%)	9/75 (12.0%)	
Pain VAS score preprocedural, median (range)	4.0 (0-10)	4 (0-10)	4 (0-7)	p=0.889§
Pain VAS score postprocedural, median (range)	2.0 (0-9)	3 (0-9)	1 (0-10)	**p=0.005**§
Pain VAS score pre- to postprocedural				p=0.118‡
Increased	13/113 (11.5%)	7/38 (18.4%)	6/75 (8.0%)	
Stable	12/113 (10.6%)	4/38 (10.5%)	8/75 (10.7%)	
Reduced	47/113 (41.6%)	19/38 (50.0%)	28/75 (37.3%	
Completely regressive	20/113 (17.7%)	3/38 (7.9%)	17/75 (22.7%	
Pain-free before and after BEST	21/113 (18.6%)	5/38 (13.2%)	16/75 (21.3%	

VAS=visual analogue scale.

‡Pearson's Chi-squared. §Mann-Whitney U test.

Supplemental Table 2. Comparison of Outcome according to Lesion Extension.

Characteristic	*Total cohort (n=113)*	*One involved anatomical area (n=95)*	*Extensive lesions (≥2 areas, n=18)*	*p-value*
Outcome in mobility				p=0.560
Decreased	10/113 (8.8%)	8/95 (8.4%)	2/18 (11.1%)	
Stable	30/113 (26.5%)	27/95 (28.4%)	3/18 (16.7%)	
Improved	48/113 (42.5%)	38/95 (40.0%)	10/18 (55.6%)	
Symptom-free	25/113 (22.1%)	22/95 (23.2%)	3/18 (16.7%)	
Outcome in aesthetic aspects				p=0.849‡
Impaired	19/113 (16.8%)	15/95 (15.8%)	4/18 (22.2%)	
Stable	21/113 (18.6%)	18/95 (18.9%)	3/18 (16.7%)	
Improved	62/113 (54.9%)	52/95 (54.7%)	10/18 (55.6%)	
Perfect	11/113 (9.7%)	10/95 (10.5%)	1/18 (5.6%)	
Pain VAS score preprocedural, median (range)	4.0 (0-10)	4.0 (0-10)	5.0 (0-10)	p=0.477§
Pain VAS score postprocedural, median (range)	2.0 (0-9)	2.0 (0-9)	2.0 (0-5)	p=0.431§
Pain VAS score pre- to postprocedural				p=0.703‡
Increased	13/113 (11.5%)	11/95 (11.6%)	2 (11.1%)	
Stable	12/113 (10.6%)	10/95 (10.5%)	2 (11.1%)	
Reduced	47/113 (41.6%)	37/95 (38.9%)	10 (55.6%)	
Completely regressive	20/113 (17.7%)	18/95 (18.9%)	2 (11.1%)	
Pain-free before and after BEST	21/113 (18.6%)	19/95 (20%)	2/18 (11.1%)	

VAS=visual analogue scale.

‡Pearson’s Chi-squared. §Mann-Whitney U test.
